# Response to olaparib in metastatic castration-resistant prostate cancer with germline *BRCA2* mutation: a case report

**DOI:** 10.1186/s12881-018-0703-9

**Published:** 2018-10-17

**Authors:** Yi Ma, Lijie He, Qianwen Huang, Shuang Zheng, Zhiqiang Zhang, Hongshi Li, Shuang Liu

**Affiliations:** 1 Department of Medical Oncology, People’s Hospital of Liaoning Province, No.33, Wenyi Road, Shenhe District, Shenyang City, 110000 China; 2 Dalian Medical University, Dalian, 116000 China

**Keywords:** Metastatic prostate cancer, BRCA2 germline mutation, PARP inhibitor, Olaparib, Liquid biopsy

## Abstract

**Background:**

Prostate cancer is a heterogeneous disease, meaning patients would benefit from different treatment strategies based on their molecular stratification. In recent years, several genomic studies have identified prostate cancers with defects in DNA repair genes. It is known that the PARP inhibitor, olaparib, has a significant synthetic lethal effect on tumors with BRCA 1/2 mutations, particularly in ovarian and breast cancer.

**Case presentation:**

In this study, we describe a patient with metastatic castration-resistant prostate cancer (mCRPC) containing a *BRCA2* germline mutation who underwent olaparib treatment. The efficacy of the treatment was monitored by serum TPSA level as well as mutation levels of circulating tumor DNA (ctDNA) using next-generation sequencing (NGS). The patient responded to the olaparib treatment as indicated by the minimal residual levels of TPSA and tumor-specific mutations of ctDNA in plasma after four months of treatment, although the patient eventually progressed at six-month post-treatment with significantly elevated and newly acquired somatic mutations in ctDNA.

**Conclusions:**

Our study provides evidence that mCRPC with *BRCA2* germline mutations could response to PARP inhibitor, which improves patient’s outcome. We further demonstrated that NGS-based genetic testing on liquid biopsy can be used to dynamically monitor the efficacy of treatment.

## Background

Germline *BRCA1/2* mutations are the greatest risk factor for inheritable breast and ovarian cancer [1]. In contrast to the diverse functions of BRCA1 in multiple DNA repair pathways and in checkpoint regulation, BRCA2 is mainly anticipated in DNA double strand breaks (DSBs) repair through RAD51-dependent homologous recombination (HR) [2]. Deleterious mutations in *BRCA2* was also implicated in a high risk of prostate cancer predisposition (8.6-fold in men ≤65 years) and more aggressiveness, as well as *BRCA1* mutations although with a much lower frequency [3–5].

Poly(ADP-ribose) polymerases (PARPs) are nuclear enzymes playing important roles in various cellular processes including DNA repair [6]. Tumor cells defective in *BRCA1/2* may rely on PAPR-dependent DNA repair, and therefore are sensitive to PARP inhibitors, which may also increase the sensitivity of tumor cells to DNA-damaging agents. Olaparib, a PARP inhibitor, has been approved by the US Food and Drug Administration (FDA) and European Medicines Agency registration for treatment of breast and ovarian cancer associated with BRCA 1/2 defects [7, 8]. Sustained responses to PARP inhibitors have also been reported in metastatic prostate cancers with DNA-repair gene mutation [9, 10]. Here we report a patient with germline *BRCA2*-mutated metastatic castration-resistant prostate cancer (mCRPC) who responded to the PARP inhibitor, olaparib.

## Case presentation

**Fig. 1:**
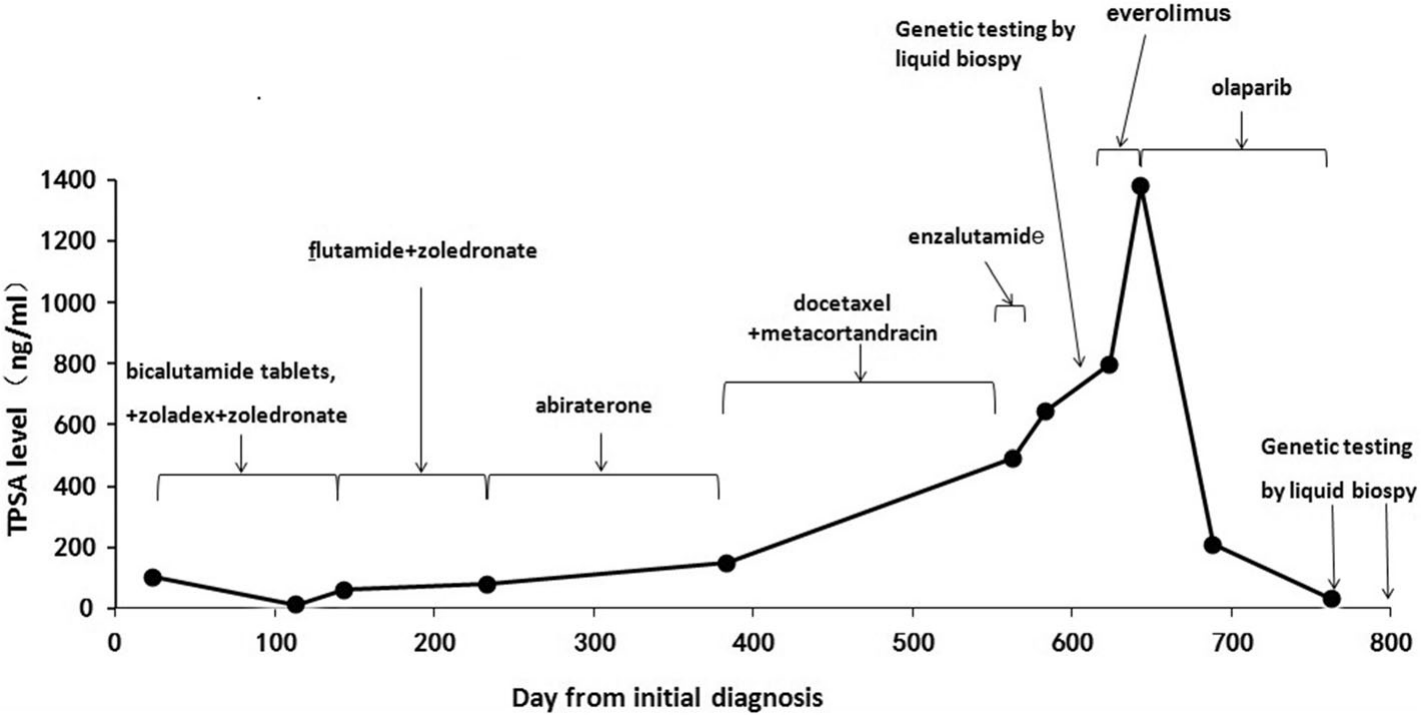
Clinical course of the patient. Serum TPSA level was measured for disease monitoring. The timeline and duration of different treatments were indicated, as well as the time points for genetic testing

The patient was a 67-year-old man who presented with dysuria. Computed tomography (CT) examination of the upper abdomen revealed multiple swollen retroperitoneal and pelvic lymph nodes and abnormal bone density on January 5th 2015. Positron Emission Tomography-CT (PET-CT) revealed hypermetabolic lesions in the left lobe of the prostate, and multiple bone sites, as well as enlarged lymph nodes of the left neck, supraclavicular region, retroperitoneal, bilateral iliac blood vessels and pelvic left side wall, which were diagnosed as malignant metastasis on February 5th 2015. A prostate biopsy was performed on February 28th 2015, and histologic assessment showed conventional adenocarcinoma with Gleason score 4 + 3 = 7, while serum TPSA level was >100 ng/mL. The clinical course of the patient was summarized in Fig. 1.

The patient started the treatment with bicalutamide tablets, zoladex and zoledronate on February 28th 2015. His TPSA level dropped to 13 ng/mL after two months of treatment, and he continued on the therapy. However, recurrent disease developed on July 9th 2015, marked by elevated TPSA up to 60 ng/mL. The patient was then switched to the treatment with flutamide and zoledronate. On October 10th 2015, due to persistent increase in TPSA level, the patient was further treated with abiraterone. On February 5th 2016, emission CT showed progression with bone metastases, with TPSA level rising to 150 ng/mL. The patient then started six cycles of systemic chemotherapy with docetaxel and metacortandracin, during which time his TPSA level continued to rise. One month after finishing the systemic chemotherapy, his TPSA level reached 492.3 ng/mL. The patient then received enzalutamide, but by August 22nd 2016, the TPSA level had risen to 644.3 ng/mL.

**Fig. 2:**
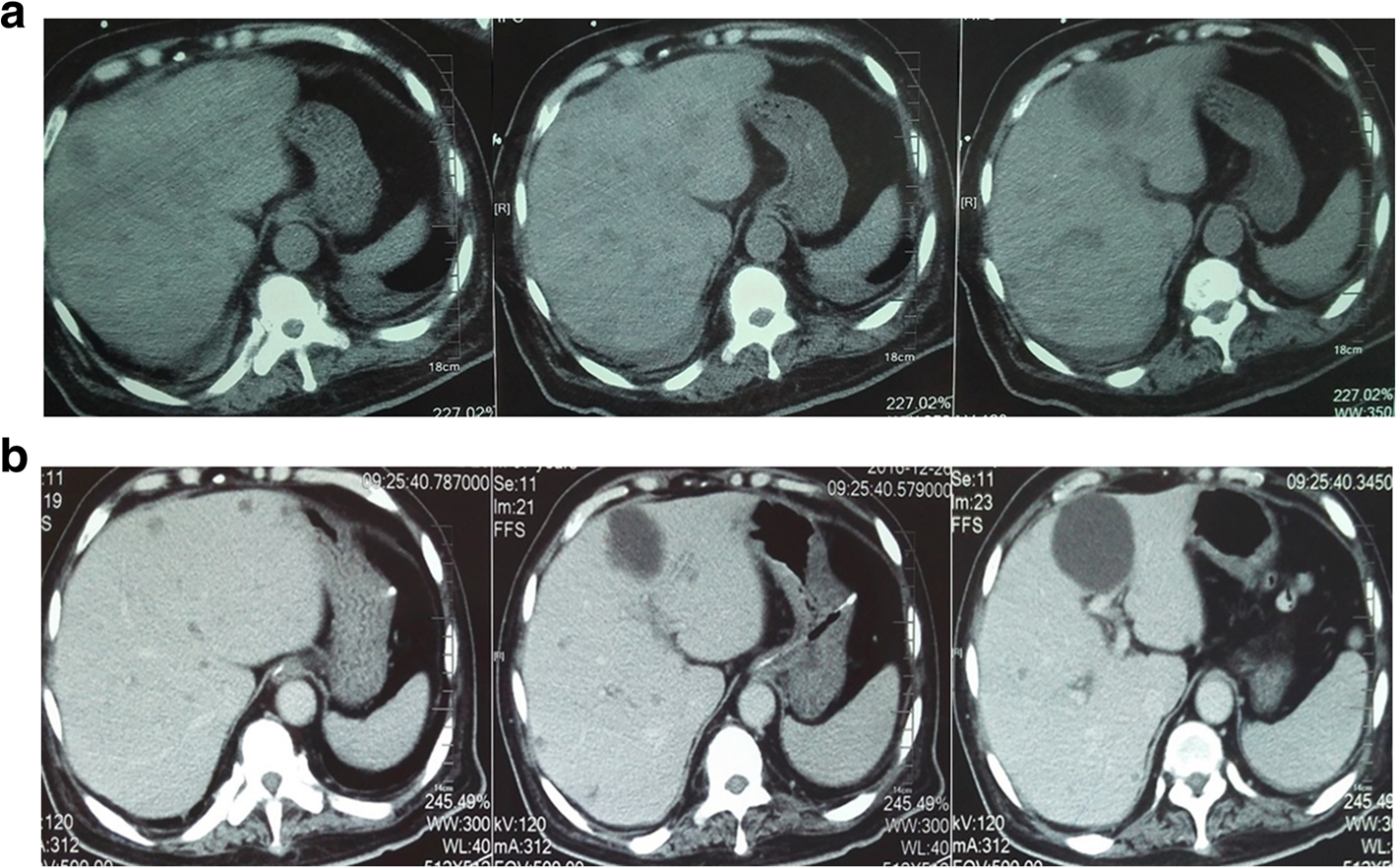
Shrinkage of the patient’s intra-hepatic lesions after two months of olaparib treatment. CT scan of the abdomen before (**a**) and after (**b**) two months of olaparib treatment

Considering the poor responses to all currently available therapies, we performed genetic testing on patient’s circulating tumor DNA (ctDNA) from blood using next-generation sequencing (NGS) targeting over 400 cancer-relevant genes. The assay was done using a commercial test. Genomic DNA from the whole blood sample was used as germline control. We detected several genomic alterations known to be associated with prostate cancer; specifically, we identified *PIK3CA*-Q546K activated mutation with a mutant allele frequency (MAF) of 17%, a TP53-DISCIFP1 fusion (MAF: 12%), 4.1 folds of relative copy number gain of the *AR* gene, as well as germline *BRCA2*-G1761X mutation. As a result, the patient started treatment with everolimus, a mTOR inhibitor, for his high MAF of *PIK3CA*-Q546K mutation. Despite this however, serum TPSA continued to increase slowly 798.9 ng/mL to 1379 ng/mL. On October 27th 2016, CT scan showed progression of multiple lymph nodes metastases, double pleural effusion and appearance of new liver metastases (Fig. 2a). The patient also developed a fever, shortness of breath and lethargy followed by unconsciousness. The patient was transferred to the intensive care unit (ICU) and underwent transfusion, respirator assisted ventilation and tracheotomy.

Due to prior detection of the *BRCA2* G1761X germline mutation and poor physical condition, the patient started on olaparib treatment, 400 mg twice daily by nasal feeding tube, on November 1st 2016; the patient tolerated the dose and his symptoms significantly relieved. On December 26th 2016, CT assessment indicated a partial response (PR) of liver metastases to olaparib (Fig. 2b). Furthermore, TPSA level was reduced from 1379 ng/mL to 208 ng/mL. Following resolution of fever, shortness of breath, lethargy and unconsciousness, the patient was transferred out of the ICU. On January 22nd 2017, the patient’s blood sample was obtained for ctDNA testing by NGS, which showed that the tumor specific mutations identified before the treatment had significantly decreased (*PIK3CA*-Q546K, 0.4%; *TP53-DISCIFP1* fusion, 0.1%; undetectable copy number gain of *AR*). After four months of the therapy, his TPSA level continued to fall to 30.65 ng/mL. However, unfortunately, the patient’s disease progressed again after six-month of the treatment, and his ctDNA testing showed that all the previous detected tumor specific mutations elevated to an even higher level compared to pretreatment (*PIK3CA*-Q546K, 19.9%; *TP53-DISCIFP1* fusion, 29.1%; 4.1 folds of relative copy number gain of *AR*), as well as a newly emerged *RB1* single copy number loss. In addition, some other somatic genomic alterations had been found in the third test (Table 1).

**Table 1:** List of the germline and somatic genomic alterations in these genetic tests

Type	Gene	Start	End	Ref	Alt	Function	NC change	AA change	AF
First genetic test									
Germline	BRCA2	chr13:32913773	chr13:32913773	G	T	stop-gained	c.G5281T	p.G1761X	N/A
Mutant	PIK3CA	chr3:178936094	chr3:178936094	C	A	missense-variant	c.C1636A	p.Q546K	17%
Second genetic test									
Germline	BRCA2	chr13:32913773	chr13:32913773	G	T	stop-gained	c.G5281T	p.G1761X	N/A
Mutant	PIK3CA	chr3:178936094	chr3:178936094	C	A	missense-variant	c.C1636A	p.Q546K	0.4%
Third genetic test									
Germline	BRCA2	chr13:32913773	chr13:32913773	G	T	stop-gained	c.G5281T	p.G1761X	N/A
Mutant	PIK3CA	chr3:178936094	chr3:178936094	C	A	missense-variant	c.C1636A	p.Q546K	19.9%
Mutant	NKX2–1	chr14:36987087	chr14:36987087	G	A	missense-variant	c.C512T	p.A171V	7.1%
Mutant	ERBB4	chr2:212587159	chr2:212587159	G	C	missense-variant	c.C842G	p.A281G	19.6%
Mutant	RUNX1	chr21:36164438	chr21:36164467	GGGCCTCCACACGGCCTCCTCCAGGCGCGC	–	inframe-deletion	c.1408_1437delGCGCGCCTGGAGGAGGCCGTGTGGAGGCCC	p.470-479del	17.6%
Mutant	NF1	chr17:29496949	chr17:29496949	G	A	missense-variant	c.G520A	p.V174I	16.6%
Mutant	MET	chr7:116412084	chr7:116412084	T	C	intron-variant	c.T3082 + 41C	N/A	6.1%
Mutant	FGFR4	chr5:176520737	chr5:176520737	C	A	missense-variant	c.C1480A	p.P494T	1.5%
Mutant	TET2	chr4:106157937	chr4:106157937	T	–	frameshift-variant	c.2838delT	p.T946 fs	0.2%
Mutant	TET2	chr4:106157939	chr4:106157939	A	C	missense-variant	c.A2840C	p.Q947P	0.2%

*chr* chromosome, *Ref* reference, *Alt* alternative, *N/A* not applicable, *NC change* nucleotide change, *AA change* amino acid change, *AF* allele frequency

## Discussion and conclusions

PARP inhibitors have proven effective in patients with breast and ovarian cancers harboring *BRCA1/2* mutations. Preliminary data also showed activity of these drugs in patients with germline BRCA1/2-mutated prostate cancer [7]. In this study, we observed a patient with germline *BRCA2* G1761X mutation as well as somatic *PIK3CA* Q546K mutation, a TP53-DISCIFP1 fusion and *AR* gene copy number gain, who had a favorable response to olaparib, although the patient eventually progressed with the emergence of olaparib resistance after six months of treatment. During the olaparib-response period, we found via liquid biopsy that the MAF of *PIK3CA* Q546K mutation decreased from 17 to 0.4%, which then increased back to 19.9% upon patient’s progression. NGS genetic testing further demonstrated that the MAF of *TP53-DISCIFP1* fusion decreased from 12 to 0.1% in response to olaparib treatment, and then increased to 29.1% when the disease progressed.

Preclinical models have suggested that PIK3CA pathway activation can alter AR transcriptional activity and lead to hormonal therapy resistance [11, 12]. A recent publication suggest that patients has longer PFS with normal PIK3CA versus those with mutation or activation [13]. This patient had poor responses to all hormonal therapies. However, the role of PIK3CA mutations in olaparib susceptibility are not currently known. We need futher research.

A recent study suggesting that outcomes to abiraterone and enzalutamide appear better in mCRPC patients harboring germline BRCA/ATM mutations (vs no mutations), but not for patients with other non-BRCA/ATM germline mutations [14]. Another recent study suggesting that men with germline and/or somatic DNA repair gene alterations may have a better response to firstline abiraterone treatment (with or without concurrent use of a PARP inhibitor) than those without mutations. This study also suggesting that patients has longer PFS with normal PTEN, TP53, and PIK3CA versus those with mutation or activation.Futher multivariable analysis including clinical and biomarker variables individually revealed DRD(DNA-damage repair defect) and TP53 as biomarkers separately associated with PFS after controlling for clinical covariates [13]. Although this patient had germline DNA repair gene alterations (BRCA2), he did not had a good response to abiraterone and a PARP inhibitor. So we suppose that the TP53 alterations perhaps dominated the tumor biology in this case and not the BRCA2 lesion. The TP53 fusion is probably pathogenic, especially if it disrupts any of the key functional domains of the p53 protein. Studies on large case series demonstrate that TP53 mutations are independent markers of bad prognosis in breast and several other cancers, and that the exact type and position of the mutation influences disease outcome [15].

In addition, when the patient’s disease progressed after the treatment of olaparib, and the ctDNA testing showed that a newly emerged *RB1* single copy number loss. RB1 alteration is rare in primary prostate adenocarcinoma [16], unlike PTEN or TP53 mutation. Previous papers have suggested that Retinoblastoma (RB1) and tumor protein 53 (TP53) tumor suppressor gene loss drives transformation of prostate adenocarcinoma (PADC) to neuroendocrine prostate cancer variants (NEPC) resistant to antiandrogen therapy (AAT) [17]. This hypothesis potentially extends beyond prostate cancer since neuroendocrine lineage transformation associated with RB1 and TP53 loss has also been observed in lung adenocarcinoma relapsing from epidermal growth factor receptor-targeted therapies [18].That may also one of the mechanisms of PARP inhibitors resistance. We need further molecular based investigantionsto identify the hypothesis.

Approximately 20% of metastatic prostate cancers harbor mutations in genes required for DNA repair by homologous recombination (HRR) such as BRCA2. HRR defects confer synthetic lethality to PARP inhibitors (PARPi) such as olaparib [19].But tumors sensitive to PARP inhibitors are known to ultimately develop resistance, so far, multiple mechanisms have been proposed. First, olaparib can trigger secondary acquired *BRCA* mutations leading to restoration of the RAD51-dependent HR pathway and allow for doublestrand breaks to undergo this less destructive repair pathway [19–22]. Intriguingly, these reversion mutations can restore the open reading frame of HR genes (e.g. BRCA2, PALB2), these have been observed not only in the setting of somatic HR mutations but also apply to germline mutations. By reverting to wild-type, such cancer cells become HR-proficient meaning that they are no longer susceptible to synthetic lethality despite ongoing PARP inhibition [23]. This patient had a germline BRCA2 p.G1761X(c.G5281 T) mutation, at the time of progression, a further test was made, but we had neither found additional somatic BRCA2 mutations nor nucleotide sequences flanking the BRCA2 original frameshift deletions, so in this case, no ORF-restoring BRCA2 mutations (i.e. reversion mutations) were discovered on the progression ctDNA analysis. Second, Cells lacking HRR must repair double-strand DNA breaks through more error-prone forms of DNA repair such as non-homologous end joining which leads to worsening mutational burden [19]. The loss of a key regulatory protein within the non-homologous end junction repair pathway, 53BP1, promotes the increased utilization of HR [24]. If both of these deficits occur in concert, then partial ATM-dependent HR repair proceeds in BRCA1- but not BRCA2-deficient cells [24, 25]. Of note, this escape mechanism has been identified clinically in BRCA1/2-associated breast cancer but may also mediate a proportion of prostate cancers that become resistant [25]. Third, upregulation of P-glycoprotein efflux transporter pumps reduces activity of many drugs, including PARP inhibitors, by depleting their intracellular availability [21, 22].

We found several new somatic mutants (i.e. NKX2–1, ERBB4, RUNX1, NF1, MET, FGFR4 and TET2) when the disease progressed, now we did not know the correlation between the somatic mutants and the resistance, but compared with the second genetic test, more new mutants had appeared, which indicate that the tumor cells were in an extremely active state and need timely treatment. In addition, these aberrations again indicate possible divergent clonal evolutionary resistance mechanisms as a result of PARP inhibition–generated selective pressures [20]. Overall, this case demonstrates that the PARP inhibitor olaparib can be effective in treating patients with germline *BRCA2* mutated prostate cancer and highlights the potential of NGS-based genetic testing on liquid biopsy as a diagnostic tool to monitor the presence and dynamics of tumor clones.

## Abbreviations

ctDNA: Circulating tumor DNA
FDA: US Food and Drug Administration
MAF: Mutant allele frequency
mCRPC: Metastatic castration-resistant prostate cancer
NGS: Next-generation sequencing
PARP: Poly-ADP-ribose polymerases
PET-CT: Positron Emission Tomography-CT
